# A pro-inflammatory diet increases the likelihood of obesity and overweight in adolescent boys: a case–control study

**DOI:** 10.1186/s13098-020-00536-0

**Published:** 2020-04-07

**Authors:** Farhad Vahid, Fatemeh Bourbour, Maryam Gholamalizadeh, Nitin Shivappa, James R. Hébert, Khatereh Babakhani, Alireza Mosavi Jarrahi, Samaneh Mirzaei Dahka, Saeid Doaei

**Affiliations:** 1 Department of Nutritional Sciences, School of Health, Arak University of Medical Sciences, Arak, Iran; 2 Department of Clinical Nutrition and Dietetics, Research Institute Shahid Beheshti University of Medical Science, Tehran, Iran; 3 Cancer Research Center, Shahid Beheshti University of Medical Sciences, Tehran, Iran; 4 Department of Epidemiology and Biostatistics, University of South Carolina, Columbia, SC USA; 5 Cancer Prevention and Control Program, University of South Carolina, Columbia, SC USA; 6 Department of Nutrition, Science and Research Branch, Islamic Azad University, Tehran, Iran; 7 School of Nursing and Midwifery, Guilan University of Medical Sciences, Rasht, Iran; 8 Research Center of Health and Environment, Guilan University of Medical Sciences, Rasht, Iran

**Keywords:** Diet, Inflammation, Obesity, Overweight, Adolescence

## Abstract

**Background:**

Obesity and Overweight at an early age can contribute with many chronic diseases such as cancers, diabetes and cardiovascular diseases. Diet-related inflammation is one of the most important underlying mechanisms that may has a key role in obesity and overweight. This paper aimed to compare the dietary inflammatory index (DII^®^) in normal weight and overweight adolescent boys.

**Methods:**

A total of 535 adolescent boys (214 cases were overweight and obese and 321 controls with normal weight) participated in this study from two schools in Tehran, Iran. The student’s weight and body composition were measured using a Bio-Impedance Analyzer (BIA) scale. A validated semi-quantitative Food Frequency Questionnaire (FFQ) was used to assess dietary inflammatory index.

**Results:**

Results obtained from modeling DII^®^ as a continuous variable identified a positive association between DII^®^ and obesity (OR = 1.08, CI 1.01–1.16). After multivariable adjustment, subjects with DII^®^ > 0.02 had at 1.5 times higher odds of obesity and overweight compared to subjects with DII^®^ ≤ 0.02 (OR = 1.52; CI 1.04–2.22).

**Conclusion:**

Our study indicated the importance of dietary-induced inflammation in the obesity and overweight during adolescence. Therefore, advising adolescent to consume diet with lower DII^®^ with more fruits and vegetables, rich sources of fiber, flavonoids, zinc, magnesium and selenium and avoiding the consumption of saturated fatty acids (SFA), trans-fatty acids, and cholesterol may support a healthy weight.

## Background

Overweight and Obesity are one of the major health problems in the world [1, 2]. The incidence of obesity in both developed and developing countries has been increasing over the past decades. This rising includes all ages, genders, and different racial and ethnic groups with various income and educational levels [3, 4]. Billions of dollars are spent annually on management and treating overweight and obesity and its associated problems [5–7]. According to global estimates and World Health Organization (WHO) reports, over 1.1 billion people in the worldwide are overweight, and of those 312 million are obese. In addition, over 155 million children are overweight and obese in the world [8–10]. Compared to average of global rates, Iran has a high prevalence of overweight and obesity [11–13]. Obesity in adolescence has an important impact on adulthood health [14, 15]. Overweight and obesity at an early age can contribute to adult obesity [16] and as well as inflammation-related non-communicable diseases such as CVD [17], mental health [18], and several types of cancer [19, 20]. Interestingly, it has been also demonstrated that dietary inflammatory index is associated with body composition in Spanish adolescents [21]. The financial and nonfinancial costs imposed by obesity on the health system of the countries are dramatically significant [5, 22–24]. Several risk factors such as genetics, lifestyle, socioeconomic status, dietary factors, and hormonal disorders have been suggested in the etiology of overweight and obesity [25, 26]. The prevention of overweight and obesity through modifying lifestyle has been considered as a cost-effective and efficient option [27, 28]. The effects of dietary habits and dietary patterns in adolescent obesity have been reported in several studies [29–33]. Numerous nutritional risk factors such as high levels of simple and total carbohydrates intake [34, 35], high intake of fat [36, 37], low intake of vegetables [38, 39], and incorrect nutritional habits, such as fast eating [40], irregular eating [41, 42], and also lack of exercise [20, 40] have been supposed to be the main factors in the etiology of overweight and obesity at adolescence.

Moreover, some studies reported that insulin resistance (IR) is one of the important factors causing adolescent overweight and obesity [43, 44]. Recent studies have reported that an inflammatory (pro-inflammatory) diet increases insulin resistance [45, 46]. To investigate the dietary inflammatory status, dietary inflammatory index (DII^®^) can be used, which is a valuable indicator that validated in various countries including Iran [47–49]. The DII^®^ is a dietary indicator developed to measure the inflammatory potential of the diet. The DII^®^ has been shown to be strongly predictive for the various inflammatory markers levels [45, 47–49]. The DII^®^ can be measured using 24-h recall interviews, food frequency questionnaires (FFQ) and food record [45, 47–49].

In the current study, we examined the association between the DII^®^ scores and overweight and obesity in adolescent boys. Our hypothesis is that a higher the DII^®^ score (indicating a pro-inflammatory diet) is associated with increased odds of overweight and obesity in adolescent boys.

## Methods

### Participants

In this case–control study, a total of 586 adolescent boys aged 12 to 16 years were enrolled from two schools in Tehran, Iran (September 2016 to October 2017). Twenty-four participants were excluded due to the lack of parental consent, 14 were excluded due to the lack of proper collaboration during the data collection period, and 12 were excluded due to the data collection difficulties (such as the BIA’s disability in measuring fat and muscle mass). Final data analysis was done on 535 students participating in the study including 214 cases who were overweight and obese students and 321 controls with normal weight (Fig. 1). The boys were recruited from the schools, which had no history of continuing education and training program on nutrition and physical activity (PA). Protocols and procedures of this study were approved by the Ethics Board of the National Nutrition and Food Technology Research Institute, Iran, and all participants provided written consents after being informed of the purpose of this research, indicating their willingness to participate.

Inclusion and exclusion criteria included volunteering to participate in the study and willingness to participate in the study and having written parental consent. The subjects were excluded if they had any diseases.

### Data collection

#### Assessment of students’ demographic status and school status

Information about the economic and social backgrounds of the students and their families was collected through a general information questionnaire. The schools’ status was also examined in terms of health-related rules, physical and social environment, welfare services, recreational, nutritional, health, and educational and extracurricular and human resources.

#### Knowledge, attitude and practice related to nutrition

Students’ knowledge and lifestyle in nutrition were assessed using five subscales of knowledge, attitude and nutrition questionnaire for adolescents, which have already been validated and proven to be reliable [50]. The questionnaire was used to evaluate the students’ nutritional knowledge about foods and their role in weight change and cholesterol level, attitudes toward healthy foods and snacks, and nutritional performance in recreational tones and nutritional misconceptions. The score of the nutrition knowledge, attitude and practice questionnaire was classified according to previous studies into 2 categories of low knowledge (less than 70% of the total score) and desired nutritional knowledge (more than or equal to 70% of the total score).

#### Assessment of nutritional status and dietary intake

The students’ nutritional information was collected via a validated food frequency questioner (FFQ). The FFQ was used to collect information about the dietary intake of the students during the last year. Energy intake was assessed using three randomly selected days of 24-hour diet recalls. This FFQ includes 168 items of foods and beverages which are mainly consumed in Iran.

### Assessment of physical activity

Information about the level of physical activity was collected using the international physical activity questionnaire (IPAQ), which has already been approved in Iran for its validity and reliability. In terms of MET (metabolic equivalent minutes) and according to the standard protocol [51], students were divided into three classes of physical activity including low activity (below 600 MET-minutes/week), moderate (between 600 and 3000 MET-minutes/week) intense activity (above 3000 MET-minutes/week).

### Assessment of the dietary inflammatory index^®^

A detailed description of the DII^®^ has been previously published [52]. To deriving inflammatory effect scores of the food parameters, the literature (approximately 2000 articles) published between 1950 and 2010 was reviewed in terms of the relationship between various micronutrients, macronutrients, and whole-food items (termed food parameters), with the inflammation. At the same time, the DII^®^ scores were standardized to a world database, which contains the means and standard deviations of food parameter intake from 11 populations around the world. The world means value for the food parameter was subtracted from the actual intake value for each food parameter, and then divided by the world standard deviation to create a z-score. In the next step, the z-scores were converted to proportions, which were then centered by doubling the value and subtracting one. This value was then multiplied by the inflammatory effect score for each food parameter. The resulted scores then were summarized across all food parameters to derive the overall DII^®^ score. More positive scores indicate more pro-inflammatory diets and more negative scores indicate more anti-inflammatory diets. In this study, 26 out of 45 food parameters were included in the calculation of the DII^®^: energy, carbohydrate, protein, total fat, alcohol, fiber, cholesterol, saturated fat, monounsaturated fat, polyunsaturated fat, omega-3, omega-6, niacin, thiamine, riboflavin, vitamin B12, vitamin B6, iron, magnesium, zinc, selenium, vitamin A, vitamin C, vitamin E, folic acid, beta carotene, caffeine.

#### Anthropometric evaluations

In this study, height, weight, body mass index (BMI), body fat percentage and muscle mass were measured as follows. The students’ height was measured using a tape meter with a precision of half a centimeter attached to the wall, in the standing position, and without shoes. The student’s weight (with a precision of 50 grams) was measured using a Bio-Impedance Analyzer (BIA) (Omron-BF511). After entering the age, sex and height of the student in BIA, the values of the BMI, body fat percentage, lean body mass percentage, and resting metabolism were determined by a trained nutritionist. The reliability and validity of this device were evaluated and confirmed in previous studies [53]. Students were categorized using the recommended z-scores of the WHO (in terms of height and BMI) and valid papers (in terms of fat and body fat percentage). WHO’s categorization of height in z-score height based on age are as follow [54]: low or short height = z2 score, high or high = over 2 + z-score, BMI by BMI for age WHO [54]: Low or lean = Zn-2 score, high or overweight = higher than 1 + z-score, high (2) or obese = higher than 2 + z-score, percentages fat based on the fat percentage chart for the age [55]: Lower = Percentile 2, High = Lower than Percentile 91, High 2 = Upper Percentage 98, Percentage of Muscle Percentage Muscle Chart for McCarthy and Associates [55]: bottom = below the percentile 2, top = higher than the percentile 98.

### Data collection method

The questionnaires were completed by a questioner while interviewing the students and their mothers. All the mothers and students were interviewed individually and the required socio-demographic information and lifestyle information was gathered. To collect information about the students’ anthropometric indicators, they were called from a list and directed by their schools’ to an interview room to be evaluated and measured. In order to increase the students and parents’ participation, the benefits of participation were described, including learning about the students’ fitness status, participating in free healthy lifestyle classes, receiving free healthy snacks and memorial gifts.

### Statistical analyses

Descriptive analyses were carried out using a t-test for continuous variables. DII^®^ (as dichotomous) was examined across the following characteristics: age, weight, height, BMI, fat mass, PA, the score of nutrition knowledge (SNK), energy, protein, total fat, carbohydrate, cholesterol, caffeine, and Self-care rating (SCR). To focus on overweight and obesity as an outcome, the DII^®^ (squared) was analyzed both as a continuous variable and a dichotomous variable, categorized based on the median value of the DII^®^ for the controls (0.02). Odds ratios (ORs) and 95% confidence intervals (CIs) for overweight and obesity as the outcome were estimated using logistic regression models, adjusting for age and total caloric intake, then fit into a model with additional adjustment for height, SNK, SCR, carbohydrates, and caffeine intake. Statistical tests were performed using SPSS. All p values were based on two-sided tests.

## Results

The DII^®^ scores in this study ranged from − 4.18 (most anti-inflammatory score) to 3.73 (most pro-inflammatory score). Table 1 shows the distribution of 214 cases of overweight and obesity and 321 normal BMI adolescences according to selected variables. The cases of overweight and obesity had higher weight, BMI, fat mass, and metabolic rate (MR), and lower age, muscle mass, and calorie intake compared to the normal BMI adolescences. The mean DII^®^ value was 1.48 (SD = 2.96) among overweight and obese and 0.99 (SD = 2.30) among the normal BMI adolescences, indicating a more pro-inflammatory diet for cases.

**Table 1:** Distribution of characteristics and dietary intakes across cases and controls

	Mean ± SD		P-value
	Cases (n = 214)	Controls (n = 321)	
Age (years)	13.95 ± 1.07	14.20 ± 1.37	0.03
Dietary inflammatory index (DII^®^)	1.48 ± 2.96	0.99 ± 2.30	0.03
Weight (kg)	82.50 ± 5.02	76.81 ± 7.54	0.04
Height (cm)	165.14 ± 5.28	165.08 ± 4.51	0.88
Body mass index (BMI)	26.40 ± 4.60	19.50 ± 2.47	< 0.0001
Fat mass	26.02 ± 6.91	14.77 ± 5.79	< 0.0001
Muscle mass	35.79 ± 3.01	39.79 ± 3.17	< 0.0001
Metabolic rate	1782.19 ± 233.87	1543.42 ± 145.51	< 0.0001
The score of nutrition knowledge	63.20 ± 4.30	63.43 ± 3.94	0.51
Energy (kcal/day)	2354.33 ± 632.64	2490.55 ± 632.49	0.01
Protein (g/day)	89.04 ± 30.25	90.24 ± 24.95	0.61
Carbohydrate (g/day)	272.93 ± 79.22	290.21 ± 71.41	0.01
Fat total (g/day)	104.51 ± 35.56	111.51 ± 40.76	0.04
Cholesterol (mg/day)	309.45 ± 229.41	302.42 ± 151.01	0.66
Caffeine (mg/day)	40.32 ± 29.55	44.55 ± 25.49	0.07
Self-care rating	44.60 ± 10.27	45.52 ± 9.17	0.28
Physical activity (MET-minutes per week)	1005 ± 71.8	1218 ± 168.7	0.76

t-test was used to compare the means

Control characteristics by the DII^®^ categories are provided in Table 2. In particular, participants in DII^®^ ≤ 0.02 categories (the DII^®^ median) had higher age, weight, MR, and BMI.

**Table 2:** Distribution of control characteristics across categories of DII^®^

	Mean ± SD		P-value
	DII^®^ ≤ 0.02 (n = 203)	DII^®^ > 0.02 (n = 118)	
Age (years)	14.35 ± 1.54	13.92 ± 0.97	< 0.0001
Weight (kg)	77.61 ± 6.58	75.44 ± 8.80	0.01
Height (cm)	165.04 ± 3.78	165.15 ± 5.56	0.83
Body mass index (BMI)	19.80 ± 2.59	18.97 ± 2.17	< 0.0001
Fat mass	15.11 ± 5.84	14.20 ± 5.67	0.17
Muscle mass	39.76 ± 3.25	39.84 ± 3.06	0.81
Metabolic rate	1555.66 ± 139.44	1522.35 ± 153.72	0.04
The score of nutrition knowledge	63.10 ± 2.36	64.00 ± 5.69	0.10
Energy (kcal/day)	2437.25 ± 302.69	2582.24 ± 960.48	0.04
Protein (g/day)	90.23 ± 14.73	90.27 ± 36.43	0.99
Carbohydrate (g/day)	286.00 ± 42.76	297.45 ± 103.47	0.16
Fat total (g/day)	107.40 ± 17.04	118.58 ± 62.96	0.06
Cholesterol (mg/day)	298.93 ± 70.22	308.42 ± 231.95	0.58
Caffeine (mg/day)	42.51 ± 14.48	48.07 ± 37.35	0.06
Self-care rating	45.11 ± 5.17	46.22 ± 13.52	0.29

t-test was used to compare the means

ORs and 95% CIs for the overweight and obesity according to continuous variables and cut-off points of the DII^®^ are shown in Table 3. Results obtained from modeling the DII^®^ (squared) as a continuous variable in relation to both overweight and obese cases identified a positive but not significant association after adjustment for age and total caloric intake (OR = 1.06 CI 0.99–1.14) and a positive and significant association after multivariate adjustment (OR = 1.08, CI 1.01–1.16). In addition, the DII^®^ (squared) expressed as a dichotomous variable and adjusting for age, and total calorie intake, subjects with the DII_®_ score > 0.02 were at 1.43 times higher odds of having OO compared to subjects with the DII_®_ ≤ 0.02 (OR_DII__®__> 0.02/≤0.02_ = 1.43 CI 1.01–2.05). After multivariable adjustment, subjects with the DII^®^ > 0.02 were at 1.52 times higher odds of having OO compared to subjects with the DII^®^ ≤ 0.02 (OR_DII®__>0.02/≤0.02_ = 1.52; CI 1.04–2.22).

**Table 3:** Odds ratios and confidence intervals for the association between DII^®^ and obesity and overweight in adolescences

	DII^®^(categorical) OR and 95% CI		P-value	DII^®^ (continuous) OR and 95% CI	P-value
	DII^®^ ≤ 0.02	DII^®^ > 0.02			
Set A	1 (ref.)	1.43 (1.01–2.05)	0.04^a^	1.06 (0.99–1.14)	0.07^a^
Set A	1 (ref.)	1.52 (1.04–2.22)	0.02^b^	1.08 (1.01–1.16)	0.02^b^
Set B	1 (ref.)	1.24 (0.76–2.03)	0.38^a^	1.00 (0.92–1.10)	0.86^a^
Set B	1 (ref.)	1.25 (0.76–2.05)	0.36^b^	1.00 (0.91–1.10)	0.90^b^
Set C	1 (ref.)	2.34 (1.50–3.66)	< 0.0001^a^	1.11 (1.03–1.21)	< 0.0001^a^
Set C	1 (ref.)	2.37 (1.50–3.74)	< 0.0001^b^	1.14 (1.04–1.24)	< 0.0001^b^
Set D	1 (ref.)	2.67 (1.49–4.75)	< 0.0001^a^	1.13 (1.01–1.26)	0.02^a^
Set D	1 (ref.)	2.67 (1.47–4.82)	< 0.0001^b^	1.11 (0.99–1.24)	0.06^b^

^a^Age and energy-adjusted

^b^Additionally adjusted for height, score of nutrition knowledge, self-care rating, carbohydrate intake, and caffeine

Set A: OR and 95% CIs for the association between DII^®^ and obesity and overweight (control = 321 case = 214)

Set B: OR and 95% CIs for the association between DII^®^ and overweight (control = 321 case = 100)

Set C: OR and 95% CIs for the association between DII^®^ and obesity (control = 321 case = 114)

Set D: OR and 95% CIs for the association between DII^®^ in overweight and obese cases (control = 100 case = 114)

In this study, we also examined obese and overweight adolescents seperately and examined their individual relationship with the DII^®^. Different results were obtained after the analysis was carried out with the DII^®^ as a continuous and dichotomous variable in relation to overweight and obese cases individually (Table 3). Results obtained from modeling the DII^®^ (squared) as a continuous variable in relation to obesity (only obese cases, set C) showed a positive and significant association after multivariable adjustment (OR_DII_^® ^ = 1.14; CI 1.04–1.24). Instead, the results obtained from modeling the DII^®^ (squared) as a continuous variable and as a dichotomous variable in relation to overweight (only overweight cases, set B) showed no significant association after adjustment for age and total caloric intake and multivariate adjustment (Table 3).

## Discussion

This case–control study was designed to investigate the relationship between dietary-induced inflammation and overweight and/or obesity in adolescent boys. As we hypothesized, inflammatory diet was associated to obesity as an inflammation-related disease.

We used DII^®^ as a valid and reliable index to study dietary-induced inflammation [46, 47]. To the best of our knowledge, this is the first study in Iran to report the relationship between diet-induced inflammation and overweight and obesity. It also seems to be the first study in the world to investigate the relationship between the DII^®^ and overweight and obesity in adolescents. Therefore, the results of our study can be considered as one of the most important parts of the relationship between diet, inflammation, obesity and related diseases. In contrast to our study, Correa-Rodríguez et al. reported that the inflammatory potential of diet, measured using the DII, is associated with obesity-related parameters such as fat-free mass and weight, but not with BMI and fat mass in adults [21]. It can be due to difference in the age of the participants. It seems that DII may be more effective on obesity-related traits during childhood and adolescence.

The analysis was performed in four different sets. When logistic regression was performed by adjusting multiple factors, there was a significant positive association between the DII^®^ and obese and overweight adolescents (set A, Table 3). Also, there was a significant relationship between the DII^®^ and obese adolescents (set C, Table 3). As well as, when overweight adolescents were considered as the control group were compared to obese adolescents as the cases, a significant association was observed between the DII^®^ and odds of obesity (set D, Table 3).

It is worth mentioning that there are few studies on the relationship between overweight and obesity and the DII^®^. In some studies [56–58], the associations between the DII^®^ with obesity and anthropometric measurements have been investigated. Ruiz- Canela M et al. [56], found a direct association between DII^®^ and obesity. They concluded that diet has a key role in the development of obesity through inflammatory modulation mechanisms [56]. Also, in a prospective cohort study, Ramallah et al. [57] reported that the DII^®^ was significantly associated with weight gain and a higher risk of developing overweight or obesity. They reported that, in a healthy population, the effect of pro- inflammatory diet was small. However, these results suggest that a pro-inflammatory diet can be a risk factor to the occurrence of obesity independently of other potential confounders such as TEI, physical activity, parental history of obesity, and baseline weight. Therefore, it is important that randomized studies investigating diet-induced inflammation to confirm this association [57]. In another study, Alam et al. [58], confirmed that there is a positive correlation between the DII^®^ and obesity, which supports their hypothesis that diet may have a role in the development of obesity in the elderly. But they admitted that it cannot be determined whether the DII^®^ is more strongly associated with visceral, subcutaneous or both types of abdominal fat mass [58]. These studies are consistent with the most important finding in our study, i.e. having a pro- inflammatory diet is related to obesity, overweight, or both of them, although based on the nature of case–control studies design, the presentation of possible mechanisms of this relationship is not possible. However, based on previous studies and proposed mechanisms, overweight and obesity is related to a low-grade inflammation resulting from chronic activation of the innate immune system, which can subsequently lead to IR, and impaired glucose tolerance [59]. Also, in a study of adolescents, CRP levels were significantly associated with overweight and obesity in both boys and girls. Whether chronic elevation of CRP levels have any direct physiologic or pathologic implication in children and/or adolescents is unclear. They suggest that during adolescence, overweight and obesity is associated with a chronic low-grade inflammatory response [60]. A systematic review has failed to found the effect of dietary consumption on biomarkers of inflammation in obese adults. However, only one out of the eight studies included in the review defined inflammation as the main outcome, and their study contained some limitations such as insufficient statistical power [61].

Similar to the study of Labonte et al. [61], after adjusting multiple factors, we did not observe a significant association between the DII^®^ and odds of being overweight while analyzing overweight adolescents as cases and conducting logistic regression models (set B, Table 3). These results and similar results indicate that for several reasons, the effect of dietary-induced inflammation in the overweight and teens with normal weight may not be significantly different. Failing to measure minor differences in blood inflammatory indicators could be one of such reasons. Previous studies found the inflammatory indices in the normal range for overweight individuals, while compared to normal people, such indices were at the highest level of rang. Therefore, a revision of laboratory tests cut points for weight and anthropometric measurements seems necessary.

Another reason for the lack of a significant difference between the effects of dietary-induced inflammation in overweight adolescents can be due to the special physiological conditions of these individuals. These physiological special conditions could be the results of the rapid growth and hormonal changes, which can affect blood inflammatory indices. However, studies with interventional designs seem to be needed to investigate the cause and effect relationship of this association and to further study the physiological and hormonal conditions.

The results of the current study should be interpreted in light of some limitations. First, the investigated relationship of this study should be tested among female adolescents. Since the physiological and hormonal conditions of boys and girls are very different especially in adolescence, other studies are recommended to investigate the association between dietary-induced inflammation and overweight and/or obesity. Second, the recall bias could be one of the limitations of case–control studies. To minimize this bias, the questionnaires were filled out by a trained nutritionist for the case and control groups. Also, adolescents’ responses were further checked by mothers to ensure their reliability. The use of valid and reliable FFQ can help in minimizing the recall bias. Third, despite the fact that the DII^®^ has been validated in terms of structural and criteria, due to lack of funding and access to facilities, this study did not measure the blood inflammatory indicators and association between those indicators with the DII^®^.

Despite such limitations, the current study has significant contributions. First, it is the first study in Iran that examines the association between dietary-induced inflammation and overweight and obesity. Furthermore, it is also the first study in the world to investigate this relationship in adolescents. However, the proper sample size and high participation rates make the findings interesting and should be taken into account, but cannot be generalized to all adolescents. generalizable to adolescents. Finally, special attention was paid to the selection of the proper control group to enhance the significance of the findings.

## Conclusion

In conclusion, preventive strategies and education can be considered as a good solution to minimize complications from obesity because, first, overweight and obesity at the adolescence is related to many diseases of older ages, and second, diet, dietary patterns, and habits have a key role in overweight and obesity at the early age. Given the fact that the results of our study indicate the importance of dietary-induced inflammation in the development of overweight and obesity, educating adolescents for the use of less inflammatory diet can help maintain their health and well-being. Therefore, advising to consume more fruits and vegetables, choosing *polyunsaturated fatty acids* (PUFA) especially omega-3 rich fishes, rich sources of vitamin A, fiber, flavonoids, vitamin B group, and minerals like zinc, magnesium, and selenium and avoiding consuming of SFA, Trans, and cholesterol can guarantee a healthy diet. Also, because of high levels of energy, SFA, Trans and salt in fast foods and junk foods, educating and advising to minimize and eliminate them from the diet of adolescents will have a significant impact on the health of communities.

## Data Availability

Not applicable.

## Abbreviations

DII: Dietary inflammatory index
FTO: Fat mass and obesity-associated gene
BMI: Body mass index
FFQ: Food Frequency Questionnaire
PA: Physical activity
SNPs: Single nucleotide polymorphisms
